# Sociodemographic and clinical factors associated with delayed diagnosis of cervical cancer: a cross-sectional study, Brazil, 2016-2020

**DOI:** 10.1590/S2237-96222025v34e20240764.en

**Published:** 2025-09-08

**Authors:** Ana Karla Monteiro Santana de Oliveira Freitas, Juliana Dantas de Araújo Santos Camargo, Amaxsell Thiago Barros de Souza, Talles Henrique de Araújo Pontes, Joyce Maria Pereira de Oliveira, Letícia Amaro Vieira, Pedro Lucas de Morais Ferreira, Sávio Ferreira Camargo, Janaina Cristiana de Oliveira Crispim Freitas

**Affiliations:** 1 Universidade Federal do Rio Grande do Norte, Natal, RN, Brazil; 2 Universidade Federal do Rio Grande do Norte, Programa de Pós-graduação em Ciências da Saúde, Natal, RN, Brazil

**Keywords:** Uterine Cervical Neoplasms, Delayed Diagnosis, Socioeconomic Factors, Comprehensive Health Care, Cross-Sectional Studies, Neoplasias del Cuello Uterino, Diagnóstico Tardío, Factores Socioeconómicos, Atención Integral de Salud, Estudios Transversales

## Abstract

**Objectives:**

To assess the time taken to diagnose cervical cancer in Brazil and identify associated sociodemographic and clinical factors in the period 2016-2020.

**Methods:**

This was a cross-sectional study of cervical neoplasms diagnosed between 2016 and 2020, using data collected from the Hospital Cancer Registry. The logistic regression model was applied to calculate odds ratios (OR) and 95% confidence intervals (95%CI). The estimates were converted to prevalence ratios (PR). A 5% significance level was adopted for all analyses.

**Results:**

A total of 23,548 cases were evaluated. Prevalence of delayed diagnosis of cervical cancer was higher in women with no formal education (PR 1.40; 95%CI 1.19; 1.65), of Black or mixed race/skin color (PR 1.15; 95%CI 1.06; 1.25), living in the Northern region (PR 1.37; 95%CI 1.21; 1.55), referred by the Brazilian National Health System (PR 1.29; 95%CI 1.18; 1.41) and with diagnosis in 2020 (PR 1.29; 95%CI 1.16; 1.43). Delayed diagnosis was less frequent among women with stage III (PR 0.31; 95%CI 0.28; 0.35) and stage IV (PR 0.37; 95%CI 0.32; 0.42) cervical cancer.

**Conclusion:**

Delayed diagnosis of cervical cancer is associated with sociodemographic inequalities and challenges faced by the Brazilian National Health System. Prevalence of delayed diagnosis was higher among Black women, women with less education and women from the Northern region. The results reinforce the need to strengthen the line of care and qualify diagnostic confirmation processes, especially for socially vulnerable populations.

## Resumen

**Objetivos:**

Evaluar el tiempo transcurrido hasta el diagnóstico de cáncer de cuello uterino en Brasil e identificar los factores sociodemográficos y clínicos asociados en el período 2016-2020.

**Métodos:**

Estudio transversal de neoplasias de cuello uterino diagnosticadas entre 2016 y 2020, utilizando datos recopilados del Registro Hospitalario de Cáncer. Se aplicó un modelo de regresión logística para calcular las razones de probabilidades (*odds ratios* - OR) y los intervalos de confianza del 95% (IC95%). Las estimaciones se convirtieron en razones de prevalencia (RP). Se adoptó un nivel de significancia del 5% para todos los análisis.

**Resultados:**

Se evaluaron 23.548 casos. La prevalencia de diagnóstico tardío de cáncer de cuello uterino fue mayor en mujeres sin educación (RP 1,40; IC95% 1,19; 1,65), raza/color de piel negro o pardo (RP 1,15; IC95% 1,06; 1,25), residentes en la región Norte (RP 1,37; IC95% 1,21; 1,55), referidas por el Sistema Único de Salud (RP 1,29; IC95% 1,18; 1,41) y con diagnóstico establecido en 2020 (RP 1,29; IC95% 1,16; 1,43). El diagnóstico tardío fue menos frecuente entre las mujeres con cáncer en estadio III (RP 0,31; IC95% 0,28; 0,35) y IV (RP 0,37; IC95% 0,32; 0,42).

**Conclusión:**

El diagnóstico tardío del cáncer de cuello uterino se asocia con desigualdades sociodemográficas y desafíos en el Sistema Único de Salud. Las mujeres negras, con menor nivel educativo y de la región norte presentaron mayor prevalencia de diagnóstico tardío. Los resultados refuerzan la necesidad de fortalecer la línea de atención y mejorar los procesos de confirmación diagnóstica, especialmente para las poblaciones socialmente vulnerables.

**Ethical aspects**

This research used public domain anonymized databases.

## Introduction

Cervical cancer remains a global challenge, despite advances in health services, especially in developing countries. In 2021, it was the fourth most commonly diagnosed form of cancer in women worldwide and the leading cause of death among women in Latin America and the Caribbean. The high mortality rates in the region, which is home to developing countries, highlight association with inequalities in terms of income, education, and access to health services (1). In Brazil, cervical cancer was the third most common type of cancer and was the second most common in the country’s North and Northeast regions in 2022. This reinforced the need for strategies aimed at screening, prevention, and early diagnosis (2).

In response to this problem, in 2020 the World Health Organization developed a global strategy aimed at preventing and reducing mortality associated with cervical cancer. The main interventions proposed include vaccination, condom use, and the implementation of screening programs aimed at early diagnosis (3). Primary prevention of human papillomavirus (HPV) begins with the vaccination of boys and girls aged 9 to 14 years, using a single-dose schedule, ideally before the onset of sexual activity.

Other preventive measures are recommended depending on the age group and context, including education on safe sexual practices, encouraging the use of condoms and making them available to sexually active individuals, male circumcision, and awareness campaigns on the risks of smoking. Tobacco use, a significant risk factor for cervical cancer and other types of cancer, often begins in adolescence. Before the age of 25, inflammatory changes caused by HPV are often detected and tend to regress (4). Therefore, the World Health Organization proposes a comprehensive approach to cancer prevention and screening. This involves a set of comprehensive, lifelong, multidisciplinary measures that incorporate elements of community education, social mobilization, vaccination, screening, treatment, and palliative care (3).

Due to the negative impacts of sociodemographic factors, Brazil has low HPV vaccination coverage rates. Economic and social factors have been associated with low vaccination coverage, including low educational level, low income, living in rural areas, and low access to information and health services. These factors are also associated with the development of cervical cancer (5). Delay in diagnosing cervical cancer can also be explained by socioeconomic inequalities, especially with regard to vaccination coverage and the development of cancer itself (6).

Diagnosis of cervical cancer is established mainly by Papanicolaou smears and colposcopy, with biopsy performed when necessary. Cervical cancer is categorized into four stages: I (localized), II (spread to nearby tissues), III (involvement of the pelvic wall and/or impaired renal function) and IV (spread to distant organs) (7).

In Brazil, screening is recommended for women and people with a uterus who have already begun sexual activity and are between 25 and 64 years old. Examinations should be performed every three years, after two consecutive normal examinations with a one-year interval (8). Secondary data recorded between 1996 and 2017 indicated that sociodemographic factors, such as age, race/skin color and geographic region, identified as obstacles to access to health services, consequently correlate with cervical cancer incidence and mortality in Brazil (9). In order to strengthen public policy initiatives aimed at the prevention and control of this form of cancer, Federal Law No. 13896/2019 established that, in cases where the main diagnostic hypothesis is malignant neoplasia, the examinations necessary for elucidation must be performed within a maximum period of 30 days (10).

The aim of this study was to evaluate the time taken until diagnosis of cervical cancer in Brazil and identify the sociodemographic and clinical factors associated with this length of time between 2016 and 2020. Understanding these aspects can favor the provision of timely treatment, considering socioeconomic inequalities and, consequently, contribute to the reduction of cervical cancer mortality rates.

## Methods

### Design

This was a cross-sectional study, intended to analyze open data made available by the Brazilian National Cancer Institute.

### Setting

Records from across Brazil were collected to assess delay in diagnosing cervical cancer in this country between 2016 and 2020.

### Participants

The study population consisted of people with malignant neoplasm of cervix uteri, code C53 of the 10^th^ revision of the International Statistical Classification of Diseases and Related Health Problems (ICD-10) (11), diagnosed between January 2016 and December 2020.

### Variables

Length of time between first consultation and diagnosis was the main variable of this study. This was categorized according to Federal Law No. 13896/2019, which established a 30-day deadline for examinations related to cancer diagnosis to be performed (10). This variable was categorized as: up to 30 days; or over 30 days (delay in cancer diagnosis).

The sociodemographic variables evaluated were: age (<30 years, 30-34, 35-39, 40-44, 45-49, 50-54, 55-59, 60-64, 65-69 and ≥70 years), schooling level (none, incomplete elementary education, complete elementary education, high school and higher education), marital status (single, married, widowed, legally separated and common law union), race/skin color (White, Black, Asian, mixed race and Indigenous), region of residence in Brazil (North, Northeast, Midwest, South and Southeast) and origin of referral (Brazilian National Health System [*Sistema Único de Saúde* - SUS], non-SUS and self-referral).

Characteristics related to diagnosis were: year of diagnosis (2016-2020), staging at diagnosis (*in situ*, I, II, III and IV) and examinations relevant for tumor diagnosis and treatment planning (anatomical pathology, tumor markers, imaging tests, clinical examination/clinical pathology and endoscopy, and exploratory surgery). Characteristics related to family history and lifestyle were also analyzed: family history of cancer (yes or no), history of alcoholic beverage consumption (never, former drinker and yes) and history of tobacco smoking (never, former smoker and yes).

### Data sources and measurement

We used data from the Hospital Cancer Registry, available through the Hospital Cancer Registry Integration Module provided by the National Cancer Institute. This is a database that stores information on diagnosis, treatment, and progression of cancer cases treated in general or specialized oncology hospitals in Brazil (12). Although it represents an important source of data for epidemiological surveillance and health planning, its coverage is not universal, being limited to cases treated in participating institutions. However, in recent years, there has been significant progress in the completeness of the recorded variables, which has contributed to improving data quality (13). The database and analysis codes used in the research are available at: https://data.scielo.org/dataset.xhtml?persistentId=doi:10.48331/scielodata.FEGGZR (14).

### Study size

All diagnoses of women living in Brazil, aged 18 years or older at the date of their first consultation, were selected for inclusion in this study. Non-analytical cases (patients who might have been treated in a hospital other than the one where initial registration occurred) and inconsistent cases, i.e., those with a diagnosis date prior to the date of the first consultation, were excluded from the analysis.

### Statistical methods

Descriptive analysis was performed using absolute and relative frequencies. Point estimates with 95% confidence intervals were used to calculate the prevalence of delayed diagnosis of cervical cancer. The chi-square test was used to analyze association between delayed diagnosis and the independent variables. The effect size for significant associations was assessed by calculating prevalence ratios (PR) and 95% confidence intervals (95%CI). The logistic regression model was applied to calculate odds ratios (OR), and the equation PR=OR/[1 + p1*(OR-1)] was used to convert these measurements (15).

The logistic regression model was also used in the multivariate analysis. The initial model was adjusted for independent variables that presented a significance level ≤0.20 in the bivariate analysis. In the second stage of the adjustment, variables with p-value <0.100 were selected. For the final model, goodness-of-fit was assessed by using the Wald chi-square test and analysis of deviance. The selected variables were those that showed significant association with the theoretical model (p-value<0.050). The bivariate and multivariate analyses were only performed for variables with missing value percentages below 35%. A 5% significance level was used for all analyses. The software used for the analyses was SPSS (Statistical Package for the Social Sciences, Chicago, United States), version 28.0.

## Results

**Figure 1:**
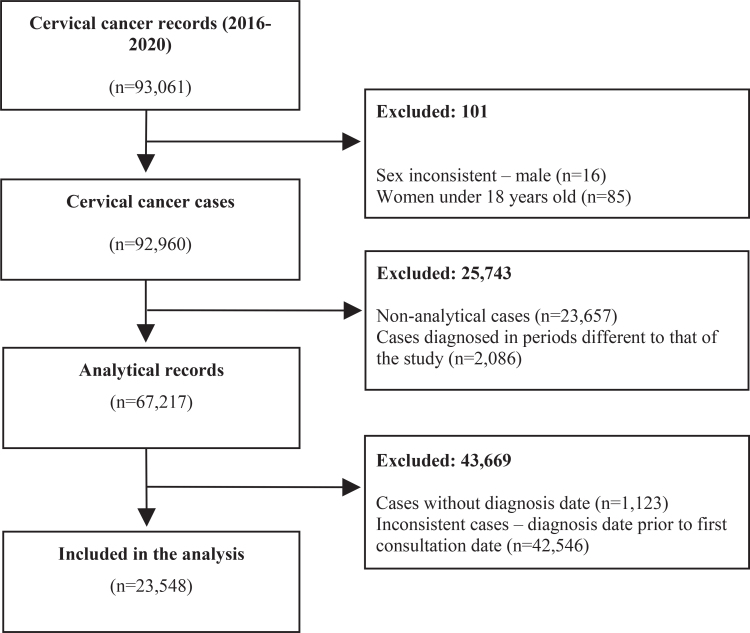
Cervical cancer case records included in the analysis. Brazil, 2016-2020

The final study sample consisted of 23,548 women diagnosed with cervical cancer (Figure 1). The highest frequencies were observed for: age under 40 years (44.7%), schooling level up to complete elementary education (63.6%), marital status = no partner (59.1%), mixed race or Black race/skin color (62.4%) and living in the South or Southeast regions of Brazil (57.4%). A considerable percentage of women were referred from the SUS network, and 2017 and 2018 were the years with the highest frequencies of diagnoses in the study period (44.5%) (Table 1).

**Table 1:** Sociodemographic and clinical characteristics of women diagnosed with cervical cancer. Brazil, 2016-2020 (n=23,548)

Sociodemographic and clinical characteristics	n (%)^a^
**Age group** (years)	23,534 (100.0)
<30	3,376 (14.3)
30-34	3,421 (14.5)
35-39	3,732 (15.9)
40-44	2,933 (12.5)
45-49	2,284 (9.7)
50-54	1,881 (8.0)
55-59	1,678 (7.1)
60-64	1,428 (6.1)
65-69	1,042 (4.4)
≥70	1,759 (7.5)
**Schooling level**	18,485^a^ (100.0)
None	1,312 (7.1)
Incomplete elementary education	6,249 (33.8)
Complete elementary education	4,201 (22.7)
High school	5,315 (28.8)
Higher education	1,408 (7.6)
**Marital status**	15,806^a^ (100.0)
Single	7,165 (45.3)
Married	5,408 (34.2)
Widowed	1,251 (7.9)
Legally separated	937 (6.0)
Common law union	1,045 (6.6)
**Race/skin color**	16,259^a^ (100.0)
White	5,919 (36.4)
Black	1,003 (6.2)
Asian	147 (0.9)
Mixed race	9,133 (56.1)
Indigenous	57 (0.4)
**Region of residence**	23,410^a^ (100.0)
North	1,394 (6.0)
Northeast	6,775 (28.9)
Midwest	1,803 (7.7)
South	4,609 (19.7)
Southeast	8,829 (37.7)
**Origin of referral**	16,208^a^ (100.0)
Brazilian National Health System (SUS)	13,997 (86.3)
Non-Brazilian National Health System	1,728 (10.7)
Self-referral	483 (3.0)
**Year of diagnosis**	23,548 (100.0)
2016	4,615 (19.6)
2017	5,237 (22.2)
2018	5,246 (22.3)
2019	4,939 (21.0)
2020	3,511 (14.9)
**Time between 1st consultation and diagnosis** (days)	23,548 (100.0)
Up to 30	15,196 (64.5)
>30	8,352 (35.5)
**Family history of cancer**	9,541^a^ (100.0)
Yes	4,270 (44.8)
No	5,271 (55.2)
**History of alcoholic beverage consumption**	9,945^a^ (100.0)
Never	7,290 (73.3)
**Former drinker**	828 (8.3)
Yes	1,827 (18.4)
**History of cigarette smoking**	11,650^a^ (100.0)
Never	7,553 (64.8)
Former smoker	1,696 (14.6)
Yes	2,401 (20.6)
**Staging at diagnosis**	17,099^a^ (100.0)
In situ	8,008 (46.8)
Stage I	2,008 (11.7)
Stage II	2,313 (13.5)
Stage III	3,196 (18.7)
Stage IV	1,574 (9.3)
**Examinations relevant for tumor diagnosis and treatment planning**	14,224^a^ (100.0)
Anatomical pathology	12,617 (88.7)
Tumor markers	1,116 (7.8)
Imaging tests	259 (1.8)
Clinical examination and clinical pathology	165 (1.2)
Endoscopy and exploratory surgery	67 (0.5)

^a^Variables with missing values.

Prevalence of delayed diagnosis of cervical cancer was 35.5% (95%CI 34.9%; 36.1%). Most women had no history of alcoholic beverage consumption or smoking. Regarding diagnosis, advanced stages (III and IV) were less frequent (28.0%) among all the stages. Anatomical pathology and tumor marker exams presented higher percentages (96.5%) for diagnosis and planning of tumor therapy (Table 1).

Statistically significant association was found between delayed diagnosis and age, schooling level, marital status, race/skin color, region of residence, origin of referral, year of diagnosis and clinical staging (p-value<0.001). The highest percentages of delayed diagnosis stood out among: younger women; those with lower schooling level; those living in the North, Northeast or Midwest regions; those of Black or mixed race/skin color; those referred by the SUS; and those with *in situ* or non-advanced clinical staging (I or II) (Table 2).

**Table 2:** Analysis of association between sociodemographic and clinical characteristics and cervical cancer diagnosis time. Brazil, 2016-2020 (n=23,548)

Characteristics	N	Diagnosis time		p-value
		<30 days	≥30 days	
**Age group** (years)				p-value<0.001
<45	13,462	8,290 (61.6)	5,172 (38.4)	
45-69	8,313	5,613 (67.5)	2,700 (32.5)	
≥70	1,759	1,281 (72.8)	478 (27.2)	
**Schooling level**				p-value<0.001
None	1,312	872 (66.5)	440 (33.5)	
Incomplete elementary education	6,249	4,044 (64.7)	2,205 (35.3)	
Complete elementary education	4,201	2,841 (67.6)	1,360 (32.4)	
High school	5,315	3,345 (62.9)	1,970 (37.1)	
Higher education	1,408	958 (68.0)	450 (32.0)	
**Marital status**				p-value<0.001
Single	7,165	4,238 (59.1)	2,927 (40.9)	
Married	5,408	3,351 (62.0)	2,057 (38.0)	
Widowed	1,251	841 (67.2)	410 (32.8)	
Legally separated	937	601 (64.1)	336 (35.9)	
Common law union	1,045	586 (56.1)	459 (43.9)	
**Race/skin color**				p-value<0.001
Black or mixed race	10,136	5,933 (58.5)	4,203 (41.5)	
Asian or indigenous	204	122 (59.8)	82 (40.2)	
White	5,919	3,899 (65.9)	2,020 (34.1)	
**Region of residence**				p-value<0.001
North	1,394	801 (57.5)	593 (42.5)	
Northeast	6,775	3,993 (58.9)	2,782 (41.1)	
Midwest	1,803	1,011 (56.1)	792 (43.9)	
South	4,609	3,075 (66.7)	1.534 (33.3)	
Southeast	8,829	6,229 (70.6)	2,600 (29.4)	
**Origin of referral**				p-value<0.001
Brazilian National Health System	13,997	8,414 (60.1)	5,583 (39.9)	
Non-Brazilian National Health System or self-referral	2.21	1,229 (71.1)	499 (28.9)	
**Year of diagnosis**				p-value<0.001
2016	4,615	3,183 (69.0)	1,432 (31.0)	
2017	5,237	3,398 (64.9)	1,839 (35.1)	
2018	5,246	3,301 (62.9)	1,945 (37.1)	
2019	4,939	3,058 (61.9)	1,881 (38.1)	
2020	3,511	2,256 (64.3)	1,255 (35.7)	
**Staging at diagnosis**				p-value<0.001
In situ	8,008	4,700 (58.7)	3,308 (41.3)	
Stage I	2,008	1,307 (65.1)	701 (34.9)	
Stage II	2,313	1,761 (76.1)	552 (23.9)	
Stage III	3,196	2,630 (82.3)	566 (17.7)	
Stage IV	1,574	1,279 (81.3)	295 (18.7)	

**Figure 2:**
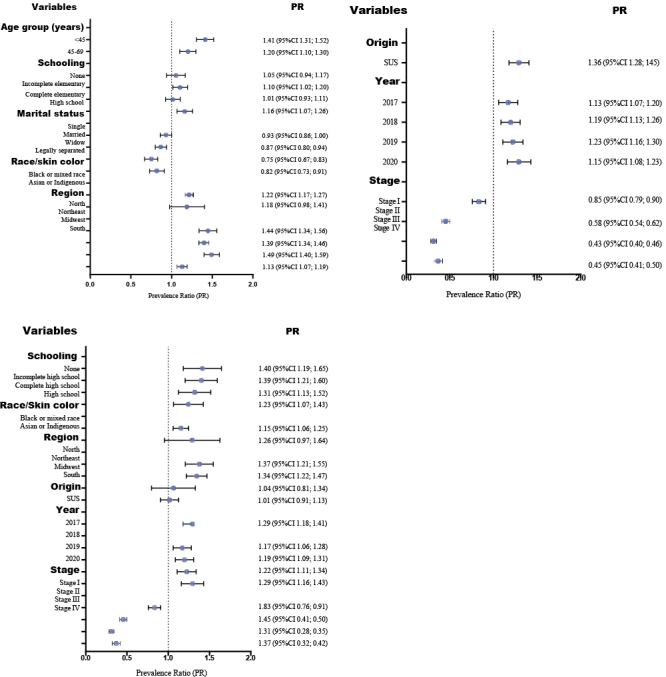
Crude and adjusted prevalence ratios (PR) and 95% confidence intervals (95%CI) of the time delay in diagnosing cervical cancer according to the study variables. Brazil, 2016-2020 (n=23,548)

The effect sizes of associations found were assessed using prevalence ratios and by adjusting all variables. Age and marital status did not retain significance and were not included in the final multivariate model (p-value>0.050) (Table 3; Figure 2). The final adjusted regression model (χ2(19)=984.71, p-value<0.001) indicated higher prevalence of delayed cervical cancer diagnosis in women with no formal education (PR 1.40; 95%CI 1.19; 1.65), those of Black or mixed race/skin color (PR 1.15; 95%CI 1.06; 1.25), those living in the Northern region (PR 1.37; 95%CI 1.21; 1.55), those referred by the SUS (PR 1.29; 95%CI 1.18; 1.41) and those with diagnosis in 2020 (PR 1.29; 95%CI 1.16; 1.43). In turn, delayed diagnosis was less frequent among women with the disease in more advanced stages, with a reduction of 69.0% in cases in stage III (PR 0.31; 95%CI 0.28; 0.35) and 63.0% in cases in stage IV (PR 0.37; 95%CI 0.32; 0.42) (Table 3; Figure 2).

**Table 3:** Crude and adjusted prevalence ratios (PR) and 95% confidence intervals (95%CI) of the time delay in diagnosing cervical cancer according to the study variables. Brazil, 2016-2020 (n=23,548)

Characteristics	Crude PR (95%CI)	Adjusted^a^ PR (95%CI)	PR of the final adjusted^b^ model (95%CI)
**Age group** (years)			
<45	1.41 (1.31; 1.52)	0.94 (0.81; 1.10)	---
45-69	1.20 (1.10; 1.30)	1.00 (0.87; 1.15)	---
≥70	Reference	Reference	
**Schooling level**			
None	1.05 (0.94; 1.17)	1.38 (1.16; 1.63)	1.40 (1.19; 1.65)
Incomplete elementary education	1.10 (1.02; 1.20)	1.37 (1.19; 1.58)	1.39 (1.21; 1.60)
Complete elementary education	1.01 (0.93; 1.11)	1.31 (1.13; 1.52)	1.31 (1.13; 1.52)
High school	1.16 (1.07; 1.26)	1.23 (1.06; 1.42)	1.23 (1.07; 1.43)
Higher education	Reference	Reference	Reference
**Marital status**			
Single	0.93 (0.86; 1.00)	0.99 (0.88; 1.10)	---
Married	0.87 (0.80; 0.94)	0.96 (0.85; 1.07)	---
Widowed	0.75 (0.67; 0.83)	0.98 (0.84; 1.13)	---
Legally separated	0.82 (0.73; 0.91)	1.08 (0.93; 1.24)	---
Common law union	Reference	Reference	
**Race/skin color**			
Black or mixed race	1.22 (1.17; 1.27)	1.14 (1.05; 1.25)	1.15 (1.06; 1.25)
Asian or indigenous	1.18 (0.98; 1.41)	1.30 (1.00; 1.69)	1.26 (0.97; 1.64)
White	Reference	Reference	Reference
**Region of residence**			
North	1.44 (1.34; 1.56)	1.36 (1.20; 1.54)	1.37 (1.21; 1.55)
Northeast	1.39 (1.34; 1.46)	1.34 (1.22; 1.47)	1.34 (1.22; 1.47)
Midwest	1.49 (1.40; 1.59)	1.02 (0.79; 1.32)	1.04 (0.81; 1.34)
South	1.13 (1.07; 1.19)	1.01 (0.90; 1.12)	1.01 (0.91; 1.13)
Southeast	Reference	Reference	Reference
**Origin of referral**			
Brazilian National Health System	1.36 (1.28; 1.45)	1.28 (1.17; 1.41)	1.29 (1.18; 1.41)
Non-Brazilian National Health System or self-referral	Reference	Reference	Reference
**Year of diagnosis**			
2016	Reference	Reference	Reference
2017	1.13 (1.07; 1.20)	1.16 (1.06; 1.28)	1.17 (1.06; 1.28)
2018	1.19 (1.13; 1.26)	1.20 (1.09; 1.31)	1.19 (1.09; 1.31)
2019	1.23 (1.16; 1.30)	1.23 (1.12; 1.36)	1.22 (1.11; 1.34)
2020	1.15 (1.08; 1.23)	1.29 (1.15; 1.43)	1.29 (1.16; 1.43)
**Staging at diagnosis**			
*In situ*	Reference	Reference	Reference
Stage I	0.85 (0.79; 0.90)	0.82 (0.75; 0.90)	0.83 (0.76; 0.91)
Stage II	0.58 (0.54; 0.62)	0.44 (0.40; 0.49)	0.45 (0.41; 0.50)
Stage III	0.43 (0.40; 0.46)	0.30 (0.27; 0.34)	0.31 (0.28; 0.35)
Stage IV	0.45 (0.41; 0.50)	0.36 (0.31; 0.41)	0.37 (0.32; 0.42)

^a^Adjustment performed for the following variables: age, schooling, marital status, race/skin color, region of residence, origin of referral, year and staging at diagnosis; ^b^Adjustment performed for the following variables: schooling, race/skin color, region of residence, origin of referral, year and staging at diagnosis.

## Discussion

This study indicated that delayed diagnosis of cervical cancer is related to sociodemographic inequalities, reflecting barriers in access to health services. Women in situations of greater vulnerability, such as those with less education, self-reporting Black or mixed race/skin color, and living in the Northern region, had a higher risk of delayed diagnosis. Being dependent on the SUS for referrals was also associated with delays, which highlighted structural challenges in the public health system. These results highlight the need for public policies aimed at reducing inequalities and improving early detection of the disease. Furthermore, delayed diagnosis is directly related to the worsening of the disease, which can result in an increase in the mortality rate of cervical cancer cases (16).

The results of this study should be interpreted considering the limitations inherent in the use of secondary databases, which may introduce biases and inaccuracies. Incomplete records are a possible limitation, since data submission by hospitals may be delayed, impacting the updating of information. The collection period may influence the interpretation of results, especially due to possible changes in diagnostic patterns in the post-pandemic period (17). Another important limitation is the impossibility of estimating the incidence and prevalence of the disease, since the data are based on institutional records and do not reflect the entire population (18).

Underreporting should also be considered, since the database used only covers hospitals that record cases on the platform, which may restrict the representativeness of the findings. Excluding the records of patients who had already been diagnosed before they arrived at the hospital, so that it was not possible to assess the time lapse since diagnosis, is also a limitation of this study. This choice, although necessary to ensure uniformity of the analysis, may have influenced the representativeness of the data, especially in the case of cervical cancer. As a consequence, the findings reflect the patterns of access to treatment, potentially underestimating the real burden of the disease in the services analyzed. Despite these limitations, the study consisted of a large number of analyzed cases, making it robust and relevant, in addition to the qualitative progress made with the Hospital Cancer Registry databases, as highlighted previously (19).

Reducing social inequalities in access to health services is crucial for effective implementation of population screening programs. Low educational level is directly associated with lower adherence to prevention programs, delayed diagnosis of cervical cancer, and reduced survival outcomes (20). This study confirms this relationship, highlighting the higher prevalence of delayed diagnosis among women with no formal education or incomplete elementary education, possibly due to limited access to information about reproductive health and socioeconomic barriers. Women with a higher educational level tend to have better access to resources, greater knowledge about health, and greater perception of risk, which favors preventive behaviors. As such, educational level plays a fundamental role in understanding the health-disease process and in seeking medical care, as demonstrated in a 2022 retrospective study conducted in the Brazilian state of Bahia, which identified an inverse association (PR 1.24; 95%CI 1.15; 1.33) between educational level and time to treatment initiation (21).

Race/skin color was a determining factor for length of time until diagnosis, with a greater delay among women who self-reported being of Black or mixed race skin color, which is in line with other studies (22,23). Black women in Brazil face significant inequalities in access to health services, which include lower screening coverage for cervical and breast cancer, greater difficulty in being referred to other services, and structural and institutional barriers that restrict access to oncological care. This reflects racial and socioeconomic inequities, which negatively affect the survival of these patients (24).

Institutional racism manifests itself in the scarcity of policies targeting this population and in their health complaints not being taken sufficiently seriously by health professionals (25), these being factors that may contribute to delayed diagnosis and delayed treatment initiation. Similar results were found in a systematic review of 45 studies, which identified ORs for late diagnosis among Black women ranging from 1.15 (95%CI 1.10; 1.20) to 1.34 (95%CI 1.15; 1.57) (22). In the United States, a study that linked national databases adjusted to exclude women who had undergone hysterectomy from the denominator, found that, between 2000 and 2012, Black women had an age-adjusted cervical cancer mortality rate more than twice that of White women (10.1 vs. 4.7 deaths per 100,000 women) (23).

Racial inequality is expressed in regional disparities, with greater delays in diagnosis in the Northern region of Brazil, where there is a higher proportion of Black and Indigenous women and limited health infrastructure (26), which hinders access to preventive examinations and specialized services. Marginalized populations face more barriers to early diagnosis, which widens health inequalities. In 2022, a study highlighted that delays in diagnosis, between 2013 and 2020, ranged from 50.0% to 70.0% in the Northern region (21). This phenomenon can be attributed to geographic characteristics, with long distances between places and a deficient transport system, which impacts access to health services. The socioeconomic conditions of the population with lower income, less education, and greater inequality in access to health services compared to other regions can also influence seeking and obtaining timely diagnosis (27).

Another relevant finding was the higher prevalence of delayed diagnosis among patients referred by the SUS. Although the SUS is essential for equity in access to health care in Brazil, the overloading of public services, difficulties in regulating specialized examinations, and long waiting lists contribute to delays in diagnosis and starting treatment. This reality disproportionately affects Black and low-income women, who depend exclusively on the SUS and often face greater difficulties in accessing the health system. The difficulties in accessing health care faced by the low-income population that uses and depends on the SUS may be an important factor in delays in diagnosis and treatment (28).

The data showed a significant increase in the prevalence of delayed diagnosis with effect from 2017, worsening in 2020 due to the COVID-19 pandemic. Although the pandemic intensified the situation, the problem had already been worsening in preceding years, reflecting factors such as underfunding of public health services, reduced investment in screening programs, and increased pent-up demand for specialized examinations and consultations. In 2020, there was a 44.6% reduction in cervical cytology tests, which may have worsened the delay in diagnosis (29). The pandemic, therefore, only accelerated the negative trend already underway, highlighting the urgent need for structural measures to reverse this situation.

Lower prevalence of delayed diagnosis was found among patients with advanced stages of the disease. This finding may be explained by the fact that, in these cases, the symptoms are already more evident, leading patients to seek medical care more urgently. However, diagnosis at an advanced stage is an indicator of inadequate access to and use of preventive screening (30).

The results of this study highlight the need for public policies to reduce racial inequalities in health services and address delayed diagnosis. There is a crucial need to expand access to screening, train professionals to eliminate discriminatory biases, and improve the flow of care in the SUS. Addressing structural racism and reversing health system shortcomings are essential for reducing inequalities in the diagnosis of cervical cancer and improving outcomes of this disease. We recommend that future studies stratify staging, procedures, and deaths according to the origin of referral (public and private) in order to provide a more detailed understanding of disparities in care and outcomes between these different contexts.

## Data Availability

The database and analysis codes used in this research are available at: https://data.scielo.org/dataset.xhtml?persistentId=doi:10.48331/scielodata.FEGGZR.
