# Supplementary material for: Sociodemographic and clinical factors associated with delayed diagnosis of cervical cancer: a cross-sectional study, Brazil, 2016-2020
Source: Epidemiol Serv Saude. 2025 Sep 8;34:e20240764. doi: 10.1590/S2237-96222025v34e20240764.en (PMC12435956; doi:10.1590/S2237-96222025v34e20240764.en)
Supplement: Translation

# Translation: Fatores sociodemográficos e clínicos associados ao atraso do diagnóstico do câncer de colo uterino: estudo transversal, Brasil, 2016-2020

**DOI:** 10.1590/S2237-96222025v34e20240764.pt

**Authors:** Ana Karla Monteiro Santana de Oliveira Freitas, Juliana Dantas de Araújo Santos Camargo, Amaxsell Thiago Barros de Souza, Talles Henrique de Araújo Pontes, Joyce Maria Pereira de Oliveira, Letícia Amaro Vieira, Pedro Lucas de Morais Ferreira, Sávio Ferreira Camargo, Janaina Cristiana de Oliveira Crispim Freitas

**Affiliations:** 1 Universidade Federal do Rio Grande do Norte, Natal, RN, Brasil; 2 Universidade Federal do Rio Grande do Norte, Programa de Pós-graduação em Ciências da Saúde, Natal, RN, Brasil

**Keywords:** Neoplasias do Colo do Útero, Diagnóstico Tardio, Fatores Socioeconômicos, Assistência Integral à Saúde, Estudos Transversais

## Abstract

**Objetivos:**

Avaliar o tempo para diagnóstico do câncer de colo uterino no Brasil e identificar os fatores sociodemográficos e clínicos associados no período 2016-2020.

**Métodos:**

Tratou-se de estudo transversal de neoplasias de colo uterino diagnosticadas entre 2016 e 2020, por meio de dados coletados do Registro Hospitalar de Câncer. Aplicou-se o modelo de regressão logística para o cálculo das razões de chances (*odds ratio*, OR) e dos intervalos de confiança de 95% (IC95%). As estimativas foram convertidas em razões de prevalências (RP). O nível de significância de 5% foi adotado para todas as análises.

**Resultados:**

Avaliaram-se 23.548 casos. A prevalência de atraso do diagnóstico de câncer de colo uterino foi maior em mulheres com nenhuma escolaridade (RP 1,40; IC95% 1,19; 1,65), raça/cor da pele preta ou parda (RP 1,15; IC95% 1,06; 1,25), residentes na região Norte (RP 1,37; IC95% 1,21; 1,55), encaminhadas pelo Sistema Único de Saúde (RP 1,29; IC95% 1,18; 1,41) e com diagnóstico estabelecido em 2020 (RP 1,29; IC95% 1,16; 1,43). O atraso no diagnóstico foi menos frequente entre mulheres com doença nos estágios III (RP 0,31; IC95% 0,28; 0,35) e IV (RP 0,37; IC95% 0,32; 0,42).

**Conclusão:**

O atraso no diagnóstico do câncer do colo do útero está associado a desigualdades sociodemográficas e desafios no Sistema Único de Saúde. Mulheres negras, com menor escolaridade e da região Norte apresentaram maior prevalência de diagnóstico tardio. Os resultados reforçam a necessidade de fortalecer a linha de cuidado e qualificar os processos de confirmação diagnóstica, especialmente para populações socialmente vulneráveis.

**Aspectos éticos**

Esta pesquisa utilizou bancos de dados de domínio público e anonimizados.

## Introdução

O câncer do colo do útero permanece como um desafio global, mesmo com o avanço dos serviços de saúde, principalmente nos países em desenvolvimento. Em 2021, foi o quarto câncer mais diagnosticado em mulheres no mundo e a principal causa de morte entre mulheres na América Latina e Caribe. As elevadas taxas de mortalidade na região, que concentra países em desenvolvimento, evidenciam a associação com desigualdades em termos de renda, educação e acesso aos serviços de saúde (1). No que diz respeito ao Brasil, o câncer do colo do útero foi o terceiro tipo de câncer mais incidente e apresentou-se como o segundo nas regiões Norte e Nordeste em 2022. Isso reforçou a necessidade de estratégias direcionadas ao rastreamento, à prevenção e ao diagnóstico precoce (2).

Em resposta a essa problemática, a Organização Mundial da Saúde elaborou, em 2020, uma estratégia global voltada para a prevenção e a redução da mortalidade associada ao câncer do colo do útero. Entre as principais intervenções propostas, destacam-se a vacinação, o uso de preservativos e a implementação de programas de rastreamento, visando ao diagnóstico precoce (3). A prevenção primária do papilomavírus humano (HPV) tem início com a vacinação de meninos e meninas de 9 a 14 anos, seguindo um esquema de dose única, idealmente antes do início da vida sexual. 

Outras medidas preventivas são recomendadas conforme a faixa etária e o contexto, o que inclui educação sobre práticas sexuais seguras, incentivo ao uso de preservativos e disponibilização destes para indivíduos sexualmente ativos, circuncisão masculina e campanhas de conscientização sobre os riscos do tabagismo. O uso do tabaco, um fator de risco significativo para o câncer do colo do útero e outros tipos de câncer, muitas vezes começa na adolescência. Antes dos 25 anos, é frequente a detecção de alterações inflamatórias pelo HPV que tendem à regressão (4). Por isso, a Organização Mundial da Saúde propõe uma abordagem abrangente para a prevenção e o rastreamento do câncer. Isso envolve o conjunto de medidas abrangentes, ao longo da vida, de forma multidisciplinar, que incorpora elementos de educação comunitária, mobilização social, vacinação, triagem, tratamento e cuidados paliativos (3).

Em decorrências dos impactos negativos de fatores sociodemográficos, o Brasil apresenta baixos índices de cobertura da vacinação contra o HPV. Fatores econômicos e sociais têm sido associados à baixa cobertura vacinal, o que inclui baixo nível educacional, baixa renda, residência em áreas rurais e baixo acesso à informação e aos serviços de saúde. Esses fatores também estão associados ao desenvolvimento do câncer do colo do útero (5). O atraso do diagnóstico do câncer do colo do útero pode ser justificado pela mesma razão que as desigualdades socioeconômicas, destacando-se na cobertura vacinal e no próprio desenvolvimento do câncer (6). 

O diagnóstico do câncer do colo do útero é estabelecido principalmente pelos exames Papanicolau e a colposcopia, com biópsia a ser realizada quando necessário. O câncer é estadiado em quatro estágios: I (localizado), II (disseminação para tecidos próximos), III (envolvimento da parede pélvica e/ou função renal comprometida) e IV (disseminação para órgãos distantes) (7).

No Brasil, o rastreamento é preconizado em mulheres e pessoas com útero que já iniciaram a atividade sexual entre os 25 e 64 anos. Os exames devem ser realizados a cada três anos, depois de dois exames consecutivos normais com intervalo de um ano (8). Dados secundários registrados entre 1996 e 2017 indicaram que fatores sociodemográficos, como idade, raça/cor da pele e região geográfica, identificados como entraves ao acesso a serviços de saúde, por consequência, apresentem correlação com a incidência e a mortalidade por câncer do colo do útero no Brasil (9). A fim de fortalecer as iniciativas das políticas públicas voltadas para a prevenção e o controle do câncer em questão, a Lei Federal nº 13.896/2019 estabeleceu que, nos casos em que a principal hipótese diagnóstica seja a de neoplasia maligna, os exames necessários à elucidação devem ser realizados no prazo máximo de 30 dias (10).

O objetivo deste estudo foi avaliar o tempo até o diagnóstico do câncer do colo do útero no Brasil e identificar os fatores sociodemográficos e clínicos associados a esse tempo entre 2016 e 2020. Compreender esses aspectos pode favorecer a oferta de tratamento oportuno, considerando as desigualdades socioeconômicas e, consequentemente, contribuir para a redução das taxas de mortalidade por câncer do colo do útero. 

## Métodos

### Delineamento do estudo

Tratou-se de estudo transversal, dirigido a analisar dados abertos disponibilizados pelo Instituto Nacional de Câncer.

### Contexto

Foram coletados os registros de todo o Brasil para avaliar o atraso no diagnóstico de câncer do colo do útero no país entre 2016 e 2020.

### Participantes

A população do estudo foi composta por pessoas com neoplasia maligna do colo do útero, código C53 da 10ª edição da Classificação Estatística Internacional de Doenças e Problemas Relacionados com a Saúde (CID-10) (11), diagnosticadas entre janeiro de 2016 e dezembro de 2020.

### Variáveis

O tempo entre a primeira consulta e o diagnóstico foi a variável principal do estudo. Esta foi categorizada conforme a Lei Federal nº 13.896/2019, que estabeleceu o prazo de 30 dias para que os exames relacionados ao diagnóstico de câncer sejam realizados (10).

Essa variável foi categorizada em: até 30 dias; ou acima de 30 dias (atraso do diagnóstico do câncer).

As variáveis sociodemográficas avaliadas foram: idade (<30 anos, 30-34, 35-39, 40-44, 45-49, 50-54, 55-59, 60-64, 65-69 e ≥70 anos), nível de escolaridade (nenhuma, ensino fundamental incompleto, ensino fundamental completo, ensino médio e ensino superior), situação conjugal (solteira, casada, viúva, separada judicialmente e união consensual), raça/cor da pele (branca, preta, amarela, parda e indígena), região de residência (Norte, Nordeste, Centro-Oeste, Sul e Sudeste) e origem do encaminhamento (Sistema Único de Saúde [SUS], não SUS e comparecimento por conta própria).

As características relacionadas ao diagnóstico foram: ano do diagnóstico (2016-2020), estadiamento no diagnóstico (*in situ*, I, II, III e IV) e exames relevantes para o diagnóstico e planejamento da terapêutica do tumor (anatomia patológica, marcadores tumorais, exames por imagem, exame clínico/patologia clínica e endoscopia e cirurgia exploradora). Características relacionadas ao histórico familiar e de estilo de vida também foram analisadas: histórico familiar de câncer (sim versus não), histórico de consumo de bebida alcoólica (nunca, ex-consumidora e sim) e histórico de consumo de tabaco (nunca, ex-consumidora e sim).

### **Fontes de dados**/mensuração

Foram utilizados dados do Registro Hospitalar de Câncer do Módulo Integrador de Registros Hospitalares de Câncer disponibilizados pelo Instituto Nacional de Câncer. Trata-se de banco de dados no qual são armazenadas informações sobre o diagnóstico, o tratamento e a evolução dos casos de neoplasias atendidos em hospitais gerais ou especializados em oncologia do Brasil (12). Embora represente importante fonte de dados para a vigilância epidemiológica e o planejamento em saúde, sua cobertura não é universal, limitando-se aos casos atendidos nas instituições participantes. Contudo, nos últimos anos, houve avanços significativos na completude das variáveis registradas, o que tem contribuído para a melhoria da qualidade dos dados (13). O banco de dados e os códigos de análise utilizados na pesquisa estão disponíveis em: https://data.scielo.org/dataset.xhtml?persistentId=doi:10.48331/scielodata.FEGGZR (14).

### Tamanho do estudo

Todos os diagnósticos de mulheres residentes no Brasil, com idade a partir de 18 anos na data da primeira consulta, foram selecionados para a inclusão no estudo. Os casos não analíticos (pacientes que poderiam ser tratados em hospital diferente de onde ocorreu o registro inicial) e os casos inconsistentes, ou seja, aqueles com data de diagnóstico anterior à data da primeira consulta, foram excluídos da análise.

### Métodos estatísticos

A análise descritiva foi realizada por meio de frequências absolutas e relativas. Realizou-se estimativa pontual com intervalos de confiança de 95% para cálculo da prevalência de ocorrência de atraso do diagnóstico de câncer do colo do útero. O teste qui-quadrado foi utilizado para analisar a associação entre o atraso do diagnóstico e as variáveis independentes. O tamanho do efeito para associações significativas foi avaliado pelo cálculo das razões de prevalências (RP) e dos intervalos de confiança de 95% (IC95%). O modelo de regressão logística foi aplicado para cálculo das razões de chances (*odds ratio*, OR), e a equação RP=OR / [1 + p1*(OR-1)] foi utilizada para conversão dessas medidas (15).

Na análise multivariada, o modelo de regressão logística também foi utilizado. O modelo inicial foi ajustado para as variáveis independentes que apresentaram nível de significância ≤0,20 na análise bivariada. Na segunda etapa do ajuste, foram selecionadas variáveis com p-valor<0,100. Para o modelo final, a qualidade do ajuste foi avaliada pelo teste qui-quadrado de Wald e pela análise da deviance. As variáveis selecionadas foram aquelas que apresentaram associações significativas com o modelo teórico (p-valor<0,050). As análises bivariada e multivariada só foram realizadas para as variáveis com percentuais de valores ausentes inferiores a 35%. O nível de significância de 5% foi adotado para todas as análises. O software utilizado para as análises foi o SPSS (Statistical Package for the Social Sciences, Chicago, Estados Unidos), versão 28.0.

## Resultados

A amostra final do estudo foi composta por 23.548 mulheres diagnosticadas com câncer do colo do útero (Figura 1). As maiores frequências foram observadas para: idades inferiores a 40 anos (44,7%), nível de escolaridade até o ensino fundamental completo (63,6%), situações conjugais sem presença de companheiro (59,1%), raças/cor da pele parda ou preta (62,4%) e regiões Sul ou Sudeste (57,4%). Um percentual expressivo de mulheres foi encaminhado da rede SUS, e 2017 e 2018 foram os anos que apresentaram as maiores frequências de diagnósticos no período do estudo (44,5%) (Tabela 1).

**Tabela 1:** Características sociodemográficas e clínicas das mulheres com diagnóstico de câncer do colo do útero. Brasil, 2016-2020 (n=23.548)

Características sociodemográficas e clínicas	n (%)^a^
**Faixa etária** (anos)	23.534 (100,0)
<30	3.376 (14,3)
30-34	3.421 (14,5)
35-39	3.732 (15,9)
40-44	2.933 (12,5)
45-49	2.284 (9,7)
50-54	1.881 (8,0)
55-59	1.678 (7,1)
60-64	1.428 (6,1)
65-69	1.042 (4,4)
≥70	1.759 (7,5)
**Nível de escolaridade**	18.485^a^ (100,0)
Nenhuma	1.312 (7,1)
Ensino fundamental incompleto	6.249 (33,8)
Ensino fundamental completo	4.201 (22,7)
Ensino médio	5.315 (28,8)
Ensino superior	1.408 (7,6)
**Situação conjugal**	15.806^a^ (100,0)
Solteira	7.165 (45,3)
Casada	5.408 (34,2)
Viúva	1.251 (7,9)
Separada judicialmente	937 (6,0)
União consensual	1.045 (6,6)
**Raça/cor da pele**	16.259^a^ (100,0)
Branca	5.919 (36,4)
Preta	1.003 (6,2)
Amarela	147 (0,9)
Parda	9.133 (56,1)
Indígena	57 (0,4)
**Região de residência**	23.410^a^ (100,0)
Norte	1.394 (6,0)
Nordeste	6.775 (28,9)
Centro-Oeste	1.803 (7,7)
Sul	4.609 (19,7)
Sudeste	8.829 (37,7)
**Origem do encaminhamento**	16.208^a^ (100,0)
Sistema Único de Saúde	13.997 (86,3)
Não Sistema Único de Saúde	1.728 (10,7)
Comparecimento por conta própria	483 (3,0)
**Ano do diagnóstico**	23.548 (100,0)
2016	4.615 (19,6)
2017	5.237 (22,2)
2018	5.246 (22,3)
2019	4.939 (21,0)
2020	3.511 (14,9)
**Tempo da 1ª consulta até o diagnóstico** (dias)	23.548 (100,0)
Até 30	15.196 (64,5)
>30	8.352 (35,5)
**Histórico familiar de câncer**	9.541^a^ (100,0)
Sim	4.270 (44,8)
Não	5.271 (55,2)
**Histórico de consumo de bebida alcoólica**	9.945^a^ (100,0)
Nunca	7.290 (73,3)
Ex-consumidora	828 (8,3)
Sim	1.827 (18,4)
**Histórico de consumo de cigarro**	11.650^a^ (100,0)
Nunca	7.553 (64,8)
Ex-consumidora	1.696 (14,6)
Sim	2.401 (20,6)
**Estadiamento no diagnóstico**	17.099^a^ (100,0)
In situ	8.008 (46,8)
Estágio I	2.008 (11,7)
Estágio II	2.313 (13,5)
Estágio III	3.196 (18,7)
Estágio IV	1.574 (9,3)
**Exames relevantes para o diagnóstico e planejamento da terapêutica do tumor**	14.224^a^ (100,0)
Anatomia patológica	12.617 (88,7)
Marcadores tumorais	1.116 (7,8)
Exames por imagem	259 (1,8)
Exame clínico e patologia clínica	165 (1,2)
Endoscopia e cirurgia exploradora	67 (0,5)

^a^Variáveis com valores ausentes.

**Figura 1:** Registros de casos de câncer do colo do útero incluídos na análise. Brasil, 2016-2020

A prevalência de ocorrência de atraso do diagnóstico de câncer do colo do útero foi 35,5% (IC95% 34,9%; 36,1%). A maior parte das mulheres não possuía histórico de consumo de bebida alcoólica nem de cigarro. Em relação ao diagnóstico, estadiamentos avançados (III e IV) foram menos frequentes (28,0%) entre todos os estágios. Exames do tipo anatomia patológica e marcadores tumorais apresentaram maiores percentuais (96,5%) para o diagnóstico e o planejamento da terapêutica do tumor (Tabela 1).

Foi verificada associação estatisticamente significativa entre atraso do diagnóstico e idade, nível de escolaridade, situação conjugal, raça/cor da pele, região de residência, origem do encaminhamento, ano do diagnóstico e estadiamento clínico (p-valor<0,001). Destacaram-se os percentuais superiores de atraso do diagnóstico em: mulheres mais jovens; com menor nível de escolaridade; residentes nas regiões Norte, Nordeste ou Centro-Oeste; de raça/cor da pele preta ou parda; encaminhadas pelo SUS; e com estadiamento clínico *in situ* ou não avançado (I ou II) (Tabela 2).

**Tabela 2:** Análise da associação entre as características sociodemográficas e clínicas e o tempo de diagnóstico de câncer do colo do útero. Brasil, 2016-2020 (n=23.548)

Características	n	Tempo do diagnóstico		p-valor
		<30 dias	≥30 dias	
**Faixa etária** (anos)				p-valor<0,001
<45	13.462	8.290 (61,6)	5.172 (38,4)	
45-69	8.313	5.613 (67,5)	2.700 (32,5)	
≥70	1.759	1.281 (72,8)	478 (27,2)	
**Nível de escolaridade**				p-valor<0,001
Nenhuma	1.312	872 (66,5)	440 (33,5)	
Ensino fundamental incompleto	6.249	4.044 (64,7)	2.205 (35,3)	
Ensino fundamental completo	4.201	2.841 (67,6)	1.360 (32,4)	
Ensino médio	5.315	3.345 (62,9)	1.970 (37,1)	
Ensino superior	1.408	958 (68,0)	450 (32,0)	
**Situação conjugal**				p-valor<0,001
Solteira	7.165	4.238 (59,1)	2.927 (40,9)	
Casada	5.408	3.351 (62,0)	2.057 (38,0)	
Viúva	1.251	841 (67,2)	410 (32,8)	
Separada judicialmente	937	601 (64,1)	336 (35,9)	
União consensual	1.045	586 (56,1)	459 (43,9)	
**Raça/cor da pele**				p-valor<0,001
Preta ou parda	10.136	5.933 (58,5)	4.203 (41,5)	
Amarela ou indígena	204	122 (59,8)	82 (40,2)	
Branca	5.919	3.899 (65,9)	2.020 (34,1)	
**Região de residência**				p-valor<0,001
Norte	1.394	801 (57,5)	593 (42,5)	
Nordeste	6.775	3.993 (58,9)	2.782 (41,1)	
Centro-Oeste	1.803	1.011 (56,1)	792 (43,9)	
Sul	4.609	3.075 (66,7)	1.534 (33,3)	
Sudeste	8.829	6.229 (70,6)	2.600 (29,4)	
**Origem do encaminhamento**				p-valor<0,001
Sistema Único de Saúde	13.997	8.414 (60,1)	5.583 (39,9)	
Não Sistema Único de Saúde ou comparecimento por conta própria	2.21	1.229 (71,1)	499 (28,9)	
**Ano do diagnóstico**				p-valor<0,001
2016	4.615	3.183 (69,0)	1.432 (31,0)	
2017	5.237	3.398 (64,9)	1.839 (35,1)	
2018	5.246	3.301 (62,9)	1.945 (37,1)	
2019	4.939	3.058 (61,9)	1.881 (38,1)	
2020	3.511	2.256 (64,3)	1.255 (35,7)	
**Estadiamento no diagnóstico**				p-valor<0,001
*In situ*	8.008	4.700 (58,7)	3.308 (41,3)	
Estágio I	2.008	1.307 (65,1)	701 (34,9)	
Estágio II	2.313	1.761 (76,1)	552 (23,9)	
Estágio III	3.196	2.630 (82,3)	566 (17,7)	
Estágio IV	1.574	1.279 (81,3)	295 (18,7)	

Os tamanhos de efeito das associações encontradas foram avaliados por meio das razões de prevalência e com o ajuste de todas as variáveis. A idade e a situação conjugal não mantiveram a significância e não entraram no modelo multivariado final (p-valor>0,050) (Tabela 3; Figura 2).

**Tabela 3:** Razão de prevalência (RP) e intervalo de confiança de 95% (IC95%) brutos e ajustados do atraso no tempo de diagnóstico de câncer do colo do útero pelas variáveis do estudo. Brasil, 2016-2020 (n=23.548)

Características	RP bruta (IC95%)	RP ajustada^a^ (IC95%)	RP do modelo final ajustado^b^ (IC95%)
**Faixa etária** (anos)			
<45	1,41 (1,31; 1,52)	0,94 (0,81; 1,10)	---
45-69	1,20 (1,10; 1,30)	1,00 (0,87; 1,15)	---
≥70	Referência	Referência	
**Nível de escolaridade**			
Nenhuma	1,05 (0,94; 1,17)	1,38 (1,16; 1,63)	1,40 (1,19; 1,65)
Ensino fundamental incompleto	1,10 (1,02; 1,20)	1,37 (1,19; 1,58)	1,39 (1,21; 1,60)
Ensino fundamental completo	1,01 (0,93; 1,11)	1,31 (1,13; 1,52)	1,31 (1,13; 1,52)
Ensino médio	1,16 (1,07; 1,26)	1,23 (1,06; 1,42)	1,23 (1,07; 1,43)
Ensino superior	Referência	Referência	Referência
**Situação conjugal**			
Solteira	0,93 (0,86; 1,00)	0,99 (0,88; 1,10)	---
Casada	0,87 (0,80; 0,94)	0,96 (0,85; 1,07)	---
Viúva	0,75 (0,67; 0,83)	0,98 (0,84; 1,13)	---
Separada judicialmente	0,82 (0,73; 0,91)	1,08 (0,93; 1,24)	---
União consensual	Referência	Referência	
**Raça/cor da pele**			
Preta ou parda	1,22 (1,17; 1,27)	1,14 (1,05; 1,25)	1,15 (1,06; 1,25)
Amarela ou indígena	1,18 (0,98; 1,41)	1,30 (1,00; 1,69)	1,26 (0.97; 1,64)
Branca	Referência	Referência	Referência
**Região de residência**			
Norte	1,44 (1,34; 1,56)	1,36 (1,20; 1,54)	1,37 (1,21; 1,55)
Nordeste	1,39 (1,34; 1,46)	1,34 (1,22; 1,47)	1,34 (1,22; 1,47)
Centro-Oeste	1,49 (1,40; 1,59)	1,02 (0,79; 1,32)	1,04 (0,81; 1,34)
Sul	1,13 (1,07; 1,19)	1,01 (0,90; 1,12)	1,01 (0,91; 1,13)
Sudeste	Referência	Referência	Referência
**Origem do encaminhamento**			
Sistema Único de Saúde	1,36 (1,28; 1,45)	1,28 (1,17; 1,41)	1,29 (1,18; 1,41)
Não Sistema Único de Saúde ou comparecimento por conta própria	Referência	Referência	Referência
**Ano do diagnóstico**			
2016	Referência	Referência	Referência
2017	1,13 (1,07; 1,20)	1,16 (1,06; 1,28)	1,17 (1,06; 1,28)
2018	1,19 (1,13; 1,26)	1,20 (1,09; 1,31)	1,19 (1,09; 1,31)
2019	1,23 (1,16; 1,30)	1,23 (1,12; 1,36)	1,22 (1,11; 1,34)
2020	1,15 (1,08; 1,23)	1,29 (1,15; 1,43)	1,29 (1,16; 1,43)
**Estadiamento no diagnóstico**			
*In situ*	Referência	Referência	Referência
Estágio I	0,85 (0,79; 0,90)	0,82 (0,75; 0,90)	0,83 (0,76; 0,91)
Estágio II	0,58 (0,54; 0,62)	0,44 (0,40; 0,49)	0,45 (0,41; 0,50)
Estágio III	0,43 (0,40; 0,46)	0,30 (0,27; 0,34)	0,31 (0,28; 0,35)
Estágio IV	0,45 (0,41; 0,50)	0,36 (0,31; 0,41)	0,37 (0,32; 0,42)

^a^Ajuste realizado para as variáveis: idade, escolaridade, situação conjugal, raça/cor da pele, região de residência, origem do encaminhamento, ano e estadiamento do diagnóstico; ^b^Ajuste realizado para as variáveis: escolaridade, raça/cor da pele, região de residência, origem do encaminhamento, ano e estadiamento do diagnóstico.

**Figura 2:** Razão de prevalências (RP) e intervalo de confiança de 95% (IC95%) brutos e ajustados do atraso no tempo de diagnóstico de câncer do colo do útero pelas variáveis do estudo. Brasil, 2016-2020 (n=23.548)

O modelo de regressão final ajustado (χ2(19)=984,71, p-valor<0,001) indicou maiores prevalências de ocorrência de atraso do diagnóstico de câncer do colo do útero em mulheres com nenhuma escolaridade (RP 1,40; IC95% 1,19; 1,65), raça/cor da pele preta ou parda (RP 1,15; IC95% 1,06; 1,25), residentes na região Norte (RP 1,37; IC95% 1,21; 1,55), encaminhadas pelo SUS (RP 1,29; IC95% 1,18; 1,41) e com diagnóstico ocorrido em 2020 (RP 1,29; IC95% 1,16; 1,43). Por sua vez, o atraso no diagnóstico foi menos frequente entre mulheres com doença em estágios mais avançados, com redução de 69,0% nos casos em estágio III (RP 0,31; IC95% 0,28; 0,35) e de 63,0% nos casos em estágio IV (RP 0,37; IC95% 0,32; 0,42) (Tabela 3; Figura 2).

## Discussão

Este estudo indicou que o atraso no diagnóstico do câncer do colo do útero está relacionado a desigualdades sociodemográficas, refletindo barreiras no acesso aos serviços de saúde. Mulheres em situação de maior vulnerabilidade, como aquelas com menor escolaridade, autodeclaradas pretas ou pardas e residentes na região Norte, apresentam maior risco de diagnóstico tardio. A dependência do SUS para encaminhamento também se mostrou associada ao atraso, o que evidenciou desafios estruturais na rede pública. Esses resultados ressaltam a necessidade de políticas públicas direcionadas para reduzir desigualdades e melhorar a detecção precoce da doença. Ademais, o atraso do diagnóstico tem relação direta com o agravamento da doença, o que pode resultar em aumento da taxa de mortalidade dos casos de câncer do colo do útero (16).

Os resultados deste estudo devem ser interpretados considerando as limitações inerentes ao uso de bases de dados secundários, que podem introduzir vieses e imprecisões. A incompletude dos registros é uma possível limitação, visto que o envio dos dados pelos hospitais pode sofrer atrasos, impactando a atualização das informações. O período de coleta pode influenciar a interpretação dos resultados, especialmente devido às possíveis alterações nos padrões de diagnóstico no período pós-pandemia (17). Outra limitação importante é a impossibilidade de estimar a incidência e a prevalência da doença, pois os dados são baseados em registros institucionais e não refletem toda a população (18).

A subnotificação também deve ser considerada, já que a base utilizada abrange apenas hospitais que aderem ao registro na plataforma, o que pode restringir a representatividade dos achados. A exclusão dos registros de pacientes que chegaram ao hospital já diagnosticadas, sem a possibilidade de avaliar o tempo desde o diagnóstico, também representa limitação do estudo. Essa escolha, embora necessária para garantir a uniformidade da análise, pode ter influenciado a representatividade dos dados, especialmente no câncer do colo do útero. Como consequência, os achados refletiram os padrões de acesso ao tratamento, potencialmente subestimando a carga real da doença nos serviços analisados. Apesar dessas limitações, o estudo apresentou grande número de casos analisados, tornando-o robusto e relevante, além dos avanços qualitativos das bases de dados do Registro Hospitalar de Câncer, já evidenciados anteriormente (19).

A redução das desigualdades sociais no acesso aos serviços de saúde é crucial para a implementação eficaz de programas de triagem populacional. A baixa escolaridade está diretamente associada à menor adesão a programas preventivos, ao diagnóstico tardio do câncer do colo do útero e aos desfechos de sobrevida reduzidos (20). Esta pesquisa confirma essa relação, evidenciando a maior prevalência de atraso no diagnóstico entre mulheres sem escolaridade ou com ensino fundamental incompleto, possivelmente devido à limitação no acesso à informação sobre saúde reprodutiva e às barreiras socioeconômicas. Mulheres com maior nível de escolaridade tendem a ter melhor acesso a recursos, maior conhecimento sobre saúde e maior percepção de riscos, o que favorece comportamentos preventivos. Assim, a escolaridade desempenha papel fundamental na compreensão do processo de saúde-doença e na busca por assistência médica, como demonstrado em um estudo retrospectivo de 2022 na Bahia, que identificou uma associação inversa (RP 1,24; IC95% 1,15; 1,33) entre o grau de escolaridade e o tempo para início do tratamento (21).

A raça/cor da pele foi fator determinante para o tempo de diagnóstico, com maior atraso entre mulheres autodeclaradas pretas ou pardas, o que está em conformidade com outros estudos (22,23). Mulheres negras no Brasil enfrentam desigualdades significativas no acesso aos serviços de saúde, o que inclui menor cobertura de rastreamento para câncer do colo do útero e mama, maior dificuldade na obtenção de encaminhamentos e barreiras estruturais e institucionais que restringem a acessibilidade ao cuidado oncológico. Isso reflete iniquidades raciais e socioeconômicas, o que afeta negativamente a sobrevida dessas pacientes (24).

O racismo institucional se manifesta na escassez de políticas direcionadas para essa população e na subvalorização de suas queixas pelos profissionais de saúde (25), fatores que podem contribuir para o atraso no diagnóstico e no início do tratamento. Resultados semelhantes foram encontrados em uma revisão sistemática com 45 estudos, que identificou OR de diagnóstico tardio em mulheres negras variando de 1,15 (IC95% 1,10; 1,20) a 1,34 (IC95% 1,15; 1,57) (22). Nos Estados Unidos, a partir da combinação de bases de dados nacionais ajustadas para excluir do denominador mulheres que haviam se submetido à histerectomia, verificou-se que, entre 2000 e 2012, mulheres negras apresentaram taxa de mortalidade por câncer do colo do útero ajustada por idade mais de duas vezes superior à de mulheres brancas (10,1 vs. 4,7 mortes por 100 mil mulheres) (23).

A desigualdade racial se expressa em disparidades regionais, com maior atraso no diagnóstico na região Norte, onde há maior proporção de mulheres negras e indígenas e infraestrutura de saúde limitada (26), o que dificulta o acesso a exames preventivos e serviços especializados. Populações marginalizadas enfrentam mais barreiras para o diagnóstico precoce, ampliando as desigualdades em saúde. Em 2022, foi destacado que o atraso no diagnóstico, entre 2013 e 2020, variou entre 50,0% e 70,0% na região Norte (21). Esse fenômeno pode ser atribuído às características geográficas, com extensas distâncias entre localidades e um sistema de transporte deficiente, o que impacta o acesso aos serviços de saúde. As condições socioeconômicas da população com menor renda, menor escolaridade e maior desigualdade no acesso à saúde em comparação com outras regiões também podem influenciar a busca e a obtenção de diagnóstico oportuno (27).

Outro achado relevante foi a maior prevalência de atraso no diagnóstico entre pacientes encaminhadas pelo SUS. Apesar de esse sistema ser fundamental para a equidade no acesso à saúde no Brasil, a sobrecarga dos serviços públicos, as dificuldades na regulação de exames especializados e as longas filas de espera contribuem para atrasos no diagnóstico e início do tratamento. Essa realidade afeta, de maneira desproporcional, mulheres negras e de baixa renda, que dependem exclusivamente do SUS e frequentemente enfrentam maior dificuldade no acesso ao sistema de saúde. As dificuldades de acesso à saúde enfrentadas pela população de baixa renda que utiliza e depende do SUS pode ser fator importante no atraso do diagnóstico e no tratamento (28).

Estes dados mostraram aumento significativo na prevalência do atraso no diagnóstico desde 2017, com agravamento em 2020 devido à pandemia de covid-19. Embora a pandemia tenha intensificado a situação, o problema já vinha se agravando nos anos anteriores, refletindo fatores como subfinanciamento da saúde pública, redução de investimentos em programas de rastreamento e aumento da demanda reprimida por exames e consultas especializadas. Em 2020, houve redução de 44,6% nos exames citopatológicos do colo do útero, o que pode ter piorado o atraso no diagnóstico (29). A pandemia, portanto, apenas acelerou a tendência negativa já em curso, destacando a necessidade urgente de medidas estruturais para reverter esse quadro.

Observou-se menor prevalência de atraso no diagnóstico entre pacientes com estágios avançados da doença. Esse achado pode ser explicado pelo fato de que, nesses casos, os sintomas já estão mais evidentes, levando as pacientes a buscarem atendimento médico com maior urgência. No entanto, o diagnóstico em estágio avançado é um indicador de acesso e utilização inadequados do rastreamento preventivo (30).

Os resultados deste estudo destacaram a necessidade de políticas públicas para reduzir as desigualdades raciais na saúde e combater o atraso no diagnóstico. É crucial ampliar o acesso ao rastreamento, capacitar profissionais para eliminar vieses discriminatórios e melhorar o fluxo de atendimento no SUS. O combate ao racismo estrutural e a reversão da insuficiência do sistema de saúde são essenciais para reduzir as desigualdades no diagnóstico do câncer do colo do útero e melhorar os desfechos dessa doença. Recomenda-se que estudos futuros estratifiquem o estadiamento, os procedimentos e os óbitos de acordo com a origem do encaminhamento (público e privado), a fim de proporcionar a compreensão mais detalhada das disparidades nos cuidados e desfechos entre esses diferentes contextos.

## Data Availability

O banco de dados e os códigos de análise utilizados na pesquisa estão disponíveis em: https://data.scielo.org/dataset.xhtml?persistentId=doi:10.48331/scielodata.FEGGZR.
